# Adapting a sexual and reproductive health program for Latina teens and their female caregivers: a qualitative study

**DOI:** 10.3389/fpubh.2025.1501757

**Published:** 2025-02-19

**Authors:** Katherine G. Merrill, Jacqueline Fuentes, Jamison Merrill, Jeff DeCelles, Jacqueline Silva, Angela Sedeño, Susana Salgado, Sara Vargas, Jennifer K. Cano, Veronica Nabor, Laura Rodriguez, Vanessa Melgoza, Corin Mora, Ana A. Baumann, Kate Guastaferro, Geri R. Donenberg

**Affiliations:** ^1^Center for Dissemination and Implementation Science, University of Illinois Chicago, Chicago, IL, United States; ^2^Floreciendo Community Advisory Council, Chicago, IL, United States; ^3^School of Public Health, University of Illinois Chicago, Chicago, IL, United States; ^4^Waves for Change, Cape Town, South Africa; ^5^The Kedzie Center, Chicago, IL, United States; ^6^Centro Romero, Chicago, IL, United States; ^7^Corazon Community Services, Cicero, IL, United States; ^8^Division of Public Health Sciences, Department of Surgery, Washington University in St. Louis, St. Louis, MO, United States; ^9^School of Global Public Health, New York University, New York, NY, United States

**Keywords:** adaptation, sexual and reproductive health, adolescence, Latina, FRAME, CBPR, mental health, intimate partner violence

## Abstract

**Background:**

Adaptation is widely recognized as important when interventions are to be delivered in new settings or with new populations. However, there are gaps in the literature on how adaptations are carried out and documented. IMARA is a 12-h evidence-based sexual health intervention for Black teens and their mothers, designed for delivery over two days. We present our systematic process of adapting IMARA for Latinas to produce the *Floreciendo* (“Blooming”) program for Latina teens (14–18 years old) and their female caregivers (e.g., mothers, sisters).

**Methods:**

Using a community-based participatory research (CBPR) approach, we carried out a qualitative study that included 7 focus groups: 4 with staff from community partner organizations (*n* = 29), 2 with Latina teens (14–18 years) (*n* = 11), and 1 with female caregivers (*n* = 5). We also conducted seven key informant interviews with experts in sexual health and Latina health. We used Escoffery’s recommended steps to guide our adaptation process. Data were thematically coded and adaptations documented using the FRAME for reporting modifications to evidence-based interventions.

**Results:**

Informed by the data, we grouped IMARA content into four sessions for Floreciendo, each with unique curricular content and designed to be delivered in two hours (eight hours total): (1) Foundations in Sexual Risk Prevention; (2) Condoms and Contraception; (3) Family Strengthening; and (4) Gender and Relationships. We documented adaptations made for each session. For example, participants emphasized unplanned pregnancy as an important issue facing Latina teens. In response, we added an activity providing hands-on experience with contraceptive methods. Participants also highlighted how gender norms and family expectations in Latine culture shape Latina teens’ sexual and reproductive health practices. We therefore developed activities and opportunities for discussion addressing these cultural influences. We removed IMARA activities considered of lower priority (e.g., portrayal of women in the media).

**Conclusion:**

This study addresses gaps in the literature by reporting in detail the adaptations we made to an evidence-based intervention using qualitative methods. The four curriculum sessions we generated through our adaptation process will form the basis of the intervention components we will test in future work using the multiphase optimization strategy (MOST) framework.

## 1 Introduction

*Adaptation* refers to thoughtful or deliberate modifications to an intervention’s content or its implementation strategies to improve their fit within a given context (1). There is wide agreement that social and behavioral interventions may require adaptation before being implemented in new settings or with new populations to achieve desired outcomes (2, 3). The science of adaptation has been cultivated since the mid-1990s (4)—particularly *cultural adaptation*, referring to systematic modifications that consider language, culture, and context to be compatible with clients’ cultural patterns, meanings, and values (5).

More recently, adaptation has gained notable attention within the field of implementation science (2, 6). This increased attention stems in part from the importance that implementation scientists place on attending to the contexts in which interventions are implemented (2). It also stems from a growing recognition that adaptations are necessary to achieve health equity (2, 7)—i.e., the principle underlying a commitment to reduce and eliminate disparities in health and social determinants to achieve the highest possible standard of health for everyone, especially those at greatest risk of poor health (8).

While program adaptation is common, there are few existing examples of adaptation processes. Documenting the adaptation process will strengthen the science of adaptation and provide much-needed guidance to others seeking to contextualize effective programs in new settings (1, 2). Detailed documentation of adaptations will provide insight into the content included in programs to support curriculum development and adaptation processes, given that access to curricula is often restricted by paywalls. It will also facilitate a collective assessment of the results of adaptations to deepen our understanding of their impact on desired outcomes of programs (3).

This study helps to fill these gaps in the literature through the Floreciendo project. Floreciendo was conceived in January 2020. The executive director of a Latine-serving community organization spoke with the first author about the need for sexual and reproductive health (SRH) programming for Latina teens in response to high rates of unplanned pregnancies in their community.

Compared to their non-Latina White counterparts, Latina teens living in the U.S. have higher rates of sexually transmitted infections (STIs) and HIV/AIDS (9), are less likely to report effectively using birth control at last sex (10), and have more than double the rate of teen pregnancy (11). Latina teens are also at risk of mental health problems (e.g., depression, anxiety) (12), intimate partner violence (IPV) (13), and substance use (14), which affect their SRH (15–18). Female caregivers (e.g., mothers, sisters, grandmothers, aunts) could serve as valuable resources for Latina teens, but SRH topics tend to be taboo in Latine households (19). One program, IMARA (Informed, Motivated, Aware, and Responsible about AIDS), has demonstrated success as a sexual health program for Black girls and their mothers. In an efficacy trial, girls who received IMARA showed a 43% reduction in incident STIs at 12-months compared to a health promotion group (20, 21).

IMARA is well-suited to being adapted for Latine families given that it seeks to address sexual health topics that are similarly taboo in a minoritized population, while focusing on the mother-daughter relationship. Effective Latina mother-daughter sexual health programs are critically needed as Latina mothers are less likely to discuss sexual risk behaviors with their teens than White or Black mothers (22, 23) but are greatly respected as figures of authority in Latine tradition (24, 25). Mother-daughter programs also align with the core Latine value of *familismo*, which emphasizes closeness within a strong family unit (26). Still, adaptations to IMARA are necessary to address the language, culture, norms, and values of Latinas and to meet the specific SRH needs of Latina teens. Furthermore, Latines are a diverse population with their own contextual challenges related to immigration, acculturation, and citizenship status, which can affect sexual health promotion (27–29).

Following the initial conversation between the executive director of the Latine-serving community organization and the first author, a partnership between community organizations, university researchers, Latina teens and their mothers, and other experts was initiated, taking a community-based participatory research (CBPR) approach (30). We first sought to understand the SRH needs of Latina teens, interest in a mother-daughter SRH program, and content that the program should include. This paper reports on how we used what we learned to adapt IMARA—the program deemed most relevant to the needs of the community—for Latina teens and their female caregivers. We will refer to the adapted program as *Floreciendo* (“Blooming” in Spanish), which is the name for the program conceived by a group of Latina teens affiliated with one of our community partner organizations.

## 2 Materials and methods

### 2.1 Community partners, council, and positionality

Floreciendo’s initial partners included The Kedzie Center, a community-funded mental health center serving residents on the Northwest side of Chicago, and Centro Romero, a community-based organization primarily serving Latine refugee and immigrant communities on the Northeast side of Chicago. In line with our CBPR approach, together, we established a Community Advisory Council, comprised of community organization representatives, university researchers, Latina teens and their mothers, and others with expertise in issues facing Latina teens. The goal of the Council was to guide the program adaptation process and the research procedures informing adaptations. Ninety percent of the Council members identified as Latina. A third organization, Corazon Community Services, expressed interest in the program and joined the team toward the tail end of the adaptation process. Corazon Community Services primarily serves the Latine community alongside others in Chicago’s Cicero and Berwyn areas.

Our team for this study included 16 co-authors, 14 of whom identify as female and 11 of whom identify as Latina. Eleven serve on Floreciendo’s Community Advisory Council and nine participated in the study as focus group participants or key informants. Eight members of our team work at or are affiliated with the community partner organizations and possess a strong understanding of the SRH needs of Latina teens. Together, we have expertise in SRH, Latina health, adolescent health, mental health, intimate partner violence, qualitative research, curriculum development, and implementation science. Recognizing that our varying identities and areas of expertise influence how we approached this research, we met and communicated regularly throughout the study to discuss the procedures, findings, curriculum adaptations, and study implications, thus enhancing the rigor of our study (31).

### 2.2 Study design

We used a qualitative study design, conducting seven focus group discussions and seven key informant interviews. We conducted 4 focus groups with staff from the community organizations (*n* = 29), 2 with Latina teens (ages 14–18) (*n* = 11), and 1 with caregivers of Latina teens (*n* = 5). Six of the focus groups were hosted between June 2022 and September 2022. The seventh focus group was hosted in October 2023 with the community organization which joined our team later on in the adaptation process.

### 2.3 IMARA overview

The original IMARA program, which draws on the social personal framework (32), is derived from three evidence-based programs: Sisters Informing Sisters about Topics on AIDS (SISTA) (33), Sistering, Informing, Healing, Living, and Empowering (SiHLE) (34), and Strengthening the Youth Life Experience (Project STYLE) (35). A community advisory board and pilot testing informed its genesis (20). The program focuses on strengthening relationships and communication between mothers and daughters, enhancing self-efficacy to use condoms, improving parental monitoring, and promoting gender empowerment and pride in Black culture. The 12-h curriculum is designed to be delivered over two days (~6 h per day), separated by one week. Separate and joint activities for mothers and daughters are interspersed. Separate activities cover parallel content, and joint activities facilitate communication practice and promote mothers’ credibility as resources for HIV/STI prevention. Activities are interactive and experiential. Participants are given homework to complete before the second workshop day. The workshop is delivered by four facilitators; two facilitators work with the daughters, two facilitators work with the mothers, and all four facilitators co-lead the joint activities. Group sizes are designed to range between about 4 and 9 dyads (20).

In consultation with the program originator and IMARA team members, the core components of the program were determined to be: (1) improving sexual health communication between teens and their female caregivers and strengthening the teen-caregiver relationship; (2) promoting cultural and gender empowerment; (3) skill-building through hands-on sexual health activities; (4) addressing foundational sexual health issues (e.g., STIs, HIV) as well as inter-related issues that affect teens’ sexual risks, like mental health and substance use; and (5) ensuring content is engaging and fun.

### 2.4 Adaptation process

**Figure 1:**
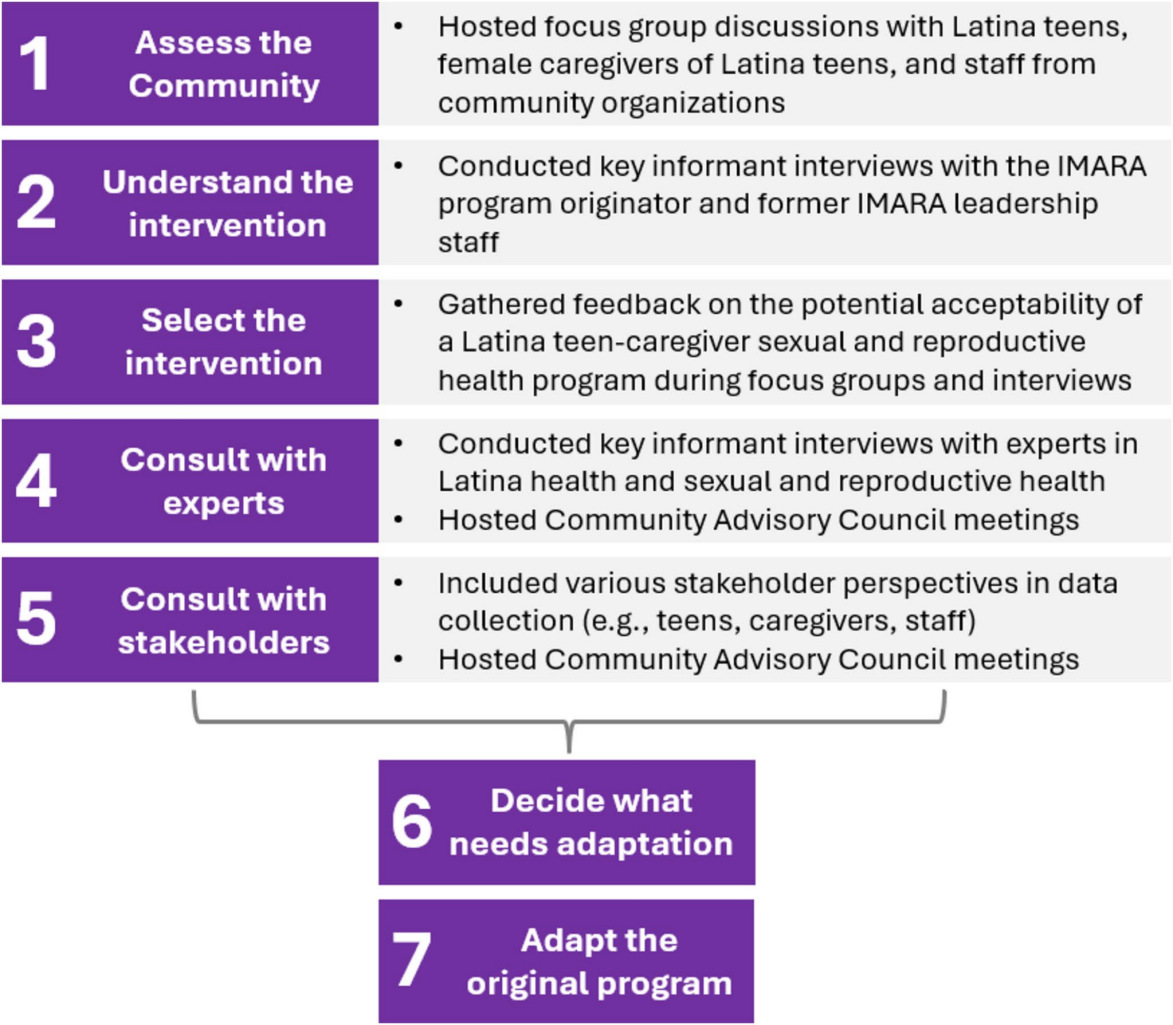
Application of Escoffery’s recommended steps for intervention adaptation (3).

We drew on a set of 11 recommended steps for intervention adaptation put forward by Escoffery et al. in a scoping review summarizing the literature on 13 adaptation frameworks (3). This paper reports on qualitative data aligning with Steps 1 through 5, some of which were conducted concurrently (Figure 1). We sought to *assess the community* (Step 1) by understanding the SRH needs of Latina teens through focus group discussions with Latina teens, female caregivers of Latina teens, and staff from community organizations. We sought to *understand the intervention* (Step 2) by interviewing the program originator and a former leadership team member of IMARA. We worked to *select the intervention* (Step 3) by gathering feedback on the potential acceptability of a SRH program for Latina teens and their female caregivers during focus groups. We *consulted with experts* in Latina health and SRH health (Step 4) during key informant interviews. We *consulted with stakeholders* (Step 5) by including a range of stakeholder perspectives in our data collection (i.e., Latina teens, female caregivers, organizational staff). We also hosted Community Advisory Council meetings to decide on our adaptation process and discuss potential adaptations. All these activities informed our *decisions about what needed adaptation* (Step 6) and our *adaptations to the original program* (Step 7).

We supplemented the qualitative data presented in this paper with other data to strengthen our adaptation process. In Step 2, we conducted a scoping review of effective SRH programs for Latine youth to determine if other mother-daughter SRH programs for Latinas already existed and to inform the content and structure of our adaptations (publication forthcoming). We also conducted a second round of focus group discussions with Latina teens, female caregivers, community organization staff, and IMARA facilitators/implementers to develop an implementation plan for the adapted program and understand considerations for its adoption and sustainability, further supporting Steps 1–5 (49).

Our adaptation reporting process is informed by the Framework for Reporting Adaptations and Modifications- Expanded (FRAME), which provides a structured approach to documenting intervention adaptations (1). The FRAME offers insights into the adaptation process, specifically: the timing and nature of adaptations, differentiation between planned and unplanned changes, identification of decision-makers, and expansion of the types of adaptations to include implementation, scale-up strategies, and content-level modifications. The FRAME also assesses fidelity consistency and the reasons behind each modification (1). Modifications are a blanket term including changes to the intervention or implementation strategies that can occur through adaptation or in an *ad hoc* manner (36).

In our adaptation process, we also prioritized the intention to optimize the intervention in future work. Our goal is to ultimately arrive at an intervention that is not only maximally effective, but also readily implementable (e.g., affordable, scalable, efficient). For this reason, we employed the multiphase optimization strategy (MOST), a translational framework for intervention development (37). The MOST framework has three phases. First, investigators explore and identify candidate intervention components, create a conceptual model for how they are hypothesized to affect the outcomes of interest, and pilot test the components during the *preparation phase*. The intervention components are then experimentally tested to inform decisions about which components should be included during the *optimization phase*. The final optimized intervention is subsequently rigorously tested during the *evaluation phase* (37). The current study activities were carried out within the *preparation phase* of MOST. We sought to define the set of intervention components—in our case, curriculum sessions—we would experimentally test in future studies to optimize the intervention.

### 2.5 Participants and procedures

To be eligible for focus groups, organizational staff had to be actively employed at one of the community organizations, 18 years or older, and an English and/or Spanish speaker. All staff from the organizations were invited to participate. Three focus groups with staff took place in person and one via Zoom.

Teens had to be between 14 and 18 years of age, identify as Latina, and an English speaker (given language limitations of the study team). Caregivers had to be either mothers or self-identified female caregivers (e.g., aunt, sister, grandmother) of Latina teens aged 14–18 and English and/or Spanish speakers. Teens and caregivers were purposively invited from an existing pool of clients at the organizations through outreach by staff. Focus groups were hosted at the community organization sites in English for teens and in Spanish for caregivers.

In line with CBPR (30), moderators and note-takers consisted of organizational staff and university researchers. Members from the organizations who supported data collection had extensive experience working with Latina teens and caregivers and many had prior qualitative research experience. All received additional training in qualitative research methods, including best practices for facilitating focus group discussions, taking notes, writing analytical memos (38), and adhering to ethical principles. Each focus group included four to eight participants, lasted ~60 to 90 min, and was audio recorded. Following the focus groups, moderators and note-takers documented their perceptions and considered emerging themes in analytical memos. Participants completed brief demographic surveys and were reimbursed $35 for their time.

Additionally, the first author conducted seven key informant interviews with individuals identified by the Community Advisory Council. This included representatives from other organizations and clinics addressing sexual and Latina health in Chicago (*n* = 3), leadership staff from the organizations (*n* = 2), the creator of IMARA (*n* = 1), and a former leadership team member of IMARA (*n* = 1). The interviews lasted 30–45 min and were hosted on Zoom. Interviewees were reimbursed $25 for their time.

### 2.6 Focus group and interview guides

Based on Escoffery’s recommended adaptation steps (3), focus group discussion guides addressed: views on being Latina; challenges Latina teens face with their SRH; strengths in the Latine community that support teens with their SRH; topics that intersect with SRH (e.g., mental health issues, intimate partner violence, substance use); sources of knowledge and support on SRH; communication between Latina teens and their mothers about SRH-related topics; and perceptions of and suggestions for a mother-daughter program for Latinas. Key informant interview guides addressed similar topics tailored to the interviewee, as well as considerations for implementation of the adapted program (the latter reported elsewhere; 49).

### 2.7 Analyses

Focus groups and interviews were transcribed verbatim and translated where needed. Transcripts were thematically coded using MAXQDA, a tool supporting qualitative data management and coding of transcripts. The first author (KM) created an initial codebook. The first and second authors (KM and JF) refined the codebook while independently coding a set of transcripts and discussing the coding process in detail. JF proceeded to code the remaining transcripts, which KM reviewed. Eleven analytical memos generated by the data collection team contributed to analyses. Preliminary findings were subjected to review and refinement through discussions with the Community Advisory Council, some of whom also participated as study participants. As such, *member checking* was used to enhance the accuracy and validity of the data (39). We sought to triangulate findings across participant types and focus groups/interviews to achieve goodness, i.e., when themes emerging authentically represent the data (39). Our collaborative, dynamic analysis process ensured a comprehensive, rigorous, and nuanced understanding of the findings to support the curriculum adaptations made (39).

One team member (JF) grouped the codes according to the FRAME. In line with the FRAME (1), we focused on modifications to the intervention’s *content*, alongside some changes made to *context*. All modifications were made *pre-implementation* and were *planned* (i.e., proactive). The *goals* of the modifications were to address cultural factors and improve the appropriateness/fit of the intervention for Latinas. The *level of delivery* targeted was intervention participants. We sought to *preserve* fidelity by frequently revisiting the core elements of IMARA (see *IMARA Overview* above) to ensure that our modifications were in alignment.

### 2.8 Ethics

This research was approved by the institutional review board at University of Illinois Chicago (IRB 00000116). We obtained verbal consent/assent from adult participants and verbal parental permission for teens who were minors (i.e., 14–17 years old). A waiver of signed consent/assent/permission was obtained from the IRB.

## 3 Results

### 3.1 Participant characteristics

Teens ranged in age from 14 to 17 years (mean = 15.7) and spanned across 9–12 grade. Most teens identified as being of Mexican origin (91%) and of second-generation immigration status (73%). All caregivers identified as being of Mexican origin and most of first-generation immigration status (80%). Caregivers reported having a high school degree or equivalent (60%), less than a high school diploma (20%) or an associate degree (20%). Most organizational staff identified as female (90%) and of Mexican descent (48%) alongside other origins (i.e., Ecuadorian, Colombian, Cuban, Puerto Rican). Most who identified as Latine also identified as being of first-generation immigration status (56%). One-quarter of staff (24%) did not identify as Latine. Most key informants identified as female (86%) and about half as Latine (56%).

### 3.2 Curriculum adaptations

#### 3.2.1 Overview

Four themes emerged from the qualitative data collected: (1) the need for basic information about SRH and related topics (e.g., mental health); (2) the importance of providing hands-on, practical experience with SRH products (e.g., condoms, contraception); (3) a desire for improving mother-daughter communication about taboo SRH topics; and (4) the influential role of gender norms and IPV on Latina teens’ SRH experiences. Informed by these themes, we adapted IMARA curriculum content to fit within four sessions for the Floreciendo curriculum: (1) Foundations in Sexual Risk Prevention; (2) Condoms and Contraception; (3) Family Strengthening; and (4) Gender and Relationships. In line with the MOST framework, we ensured that each of the three sessions following the Foundations session (i.e., the sessions we planned to test in a future optimization trial) could stand alone and be delivered in any order. To do so, we removed the homework assignments from IMARA and wrote the curriculum such that themes addressed in the three sessions would be distinct from each other and build only on themes covered in the Foundations session—not on themes in the other sessions.

Table 1 summarizes the resulting content areas of each Floreciendo session and the nature of the modifications made to the IMARA content using the FRAME. Appendices 1–4 provide further detail on the modifications made. Below, we describe the participants’ feedback according to each of IMARA’s sessions and the modifications made from the original IMARA program to Floreciendo.

**Table 1:** Summary of adaptations to IMARA content by Floreciendo session using FRAME (1).

Foundations in sexual risk behavior			
WHAT is adapted? Content areas:	What is the NATURE of the adaptation?	DESCRIPTION of the adaptation (examples)	Feedback from WHOM informed the adaptation?
Mental health (affect management)	Adding elements/tailoring/refinement	Added an explanation of what mental health is and how the feeling thermometer activity can promote mental health.	Teens, Staff, Key informants
STIs and HIV	Adding elements/tailoring/refinement /removing elements	Removed statistics (“About 3 million teens in the US get an STI every year”).	Teens, Caregivers, Staff, Key informants
Risky sexual behavior, including alcohol use	Adding elements/tailoring/refinement	Added time for teen facilitators to share a personal story about a risky situation they faced.	Teens, Staff, Key Informants
Adolescent development and parental monitoring (caregivers only)	Tailoring/refinement	Refined such that caregivers walk through the creation of a parental monitoring plan together instead of working on one individually.	None
Reproductive system (including anatomy, menstruation)	Adding elements	Created anatomy handouts for a person with a vagina and with a penis.	Teens, Caregivers, Staff, Key informants
Sexual pleasure	Adding elements	Added discussion about sexual pleasure as a primary reason people have sex.	Teens, Caregivers, Staff, Key informants
Human papillomavirus (HPV)	Adding elements	Added a true/false statement about what HPV is and that there is a vaccine to prevent HPV.	Key informants

Condoms and contraception			
WHAT is adapted? Content areas:	What is the NATURE of the adaptation?	DESCRIPTION of the adaptation (examples)	Feedback from WHOM informed the adaptation?
External condoms	Tailoring/refinement	Changed wording from “condom” to “external condom.”	Teens, Staff, Key informants
Internal condoms	Tailoring/refinement/adding elements	Changed wording from “female condom” to “internal condom.” Added space for participants to practice using internal condoms.	Teens, Staff, Key informants
Dental dams	Tailoring/refinement	Separated the explanation of what a dental dam is and a demonstration of how to use one into its own activity.	Teen, Caregivers, Staff
Contraception	Adding elements	Added a discussion about pregnancy, a demonstration of contraceptive methods, and an explanation of emergency contraception.	Teens, Caregivers, Staff, Key informants

Family strengthening			
WHAT is adapted? Content areas:	What is the NATURE of the adaptation?	DESCRIPTION of the adaptation (examples)	Feedback from WHOM informed the adaptation?
Communication styles for teens and caregivers	Tailoring/refinement	Clarified definitions for aggressive and passive communication styles in contrast to assertive communication.	Teens, Caregivers, Staff, Key informants
Role playing teen/caregiver communication	Tailoring/refinement	Made minor updates to how the role plays are carried out.	Key informant
Latine family norms and expectations	Adding elements	Added an activity for teens and caregivers to take turns sharing views about being a Latina teen/caregiver, expectations in Latine families, and how their teens/caregivers can support them.	Teens, Caregivers, Staff, Key informants

Gender and relationships			
WHAT is adapted? Content areas:	What is the NATURE of the adaptation?	DESCRIPTION of the adaptation (examples)	Feedback from WHOM informed the adaptation?
Healthy/unhealthy relationships	Tailoring/refinement/removing elements	Modified the activity such that participants decide whether relationship scenarios are healthy or unhealthy in small groups rather than individually.	Teens, Staff, Key informants
Partner communication	Tailoring/refinement/removing elements	Kept one role play but removed three role plays for time.	Staff
Intimate partner violence	Adding elements/tailoring/refinement	Changed wording from “abuse” to “violence.” Customized handouts on resources for IPV.	Teens, Staff
Consent	Adding elements	Added content on consent and sexual consent and what these terms mean.	Teens, Staff
Gender identities and sexual orientation	Adding elements	Added an activity to introduce different gender identities, personal pronouns, and various sexual orientations.	Teens, Staff, Key informants
Gender norms and relationships in Latine culture	Adding elements	Added discussion about the lyrics for a popular song, *El Toxico*, and how they reflect gender norms in Latine culture.	Teens, Staff, Key informants

Other FRAME reporting categories apply to all modifications in the table as follows: (1) WHEN did the modification occur? Pre-implementation; (2) Were adaptations PLANNED or UNPLANNED? Planned/proactive; (3) What was the GOAL? To address cultural factors and improve the appropriateness/fit of the intervention for Latinas; (4) At what LEVEL OF DELIVERY (for whom/what is the modification made)? Intervention participants; (5) Relationship to FIDELITY or core elements? Fidelity preserved. See Appendices 1–4 for full descriptions of adaptations made.

#### 3.2.2 Session 1: Foundations in sexual risk prevention

Focus group participants and key informants described how both Latina teens and their female caregivers lack basic information about SRH. Teens reported having questions about SRH but usually turning to friends, even though they recognized their friends as being unreliable sources of information. Many female caregivers never received formal education about SRH, in some cases because they did not complete school before immigrating to the U.S. As a key informant explained, *“A lot of Hispanic women are not taught about sex, STIs, and how to prevent pregnancy. They’re in the same boat as the teens, in a sense.”*

Participants raised foundational issues to address in the curriculum, including STIs, HIV, oral and anal sex, and responsible sexual decision-making. They recommended addressing common myths in Latine households—e.g., that using tampons will *“make you want to have sex”* (staff focus group). They wanted information about preventing unplanned pregnancy, which was perceived as a common issue among Latina teens: *“What are the signs of pregnancy? What can cause pregnancy and how can we not get pregnant?”* (teen focus group). They requested information about reproductive anatomy (including the clitoris) and sexual pleasure. For example:

*Having a better understanding of their body, having language for things, being able to acknowledge pleasure—all of these may be concepts that [teens and caregivers] do not have the language or permission to talk about. If teens do not know their own bodies, it’s going to be very hard for them to be empowered to give [sexual] consent* (Key informant).

Participants also wanted information about mental health problems and substance use, which they perceived as important and interrelated with sexual health and behavior. As a teen explained, *“Depression is really common in Latinos and can lead people to neglect their SRH.”* Key informants recommended addressing human papillomavirus (HPV) infection, given that it is relatively unknown in the community. Participants further requested a list of resources for SRH services and information (e.g., locations, websites).

##### 3.2.2.1 Curriculum modifications

We made minor modifications to IMARA activities addressing the basics about STIs/HIV (e.g., how they are spread) and risky sexual situations for clarity. For example, we integrated definitions for key terms, like condoms and masturbation. We added a definition for mental health and clarified how it interlinks with an existing activity addressing emotion regulation (the “Feeling Thermometer”). We created new content on risks for unplanned pregnancy, sexual pleasure, and HPV and clarified myths in Latine culture (e.g., regarding condom use). We created handouts on reproductive anatomy, updated the format of existing handouts for visual appeal, and generated up-to-date community resource handouts with maps of available services (Appendix 1).

#### 3.2.3 Session 2: Condoms and contraception

Participants requested information about various types of condoms (e.g., internal condoms), since they explained that many Latinas are only aware of external condoms. They also highlighted the importance of addressing forms of contraception, since for many Latinas the Catholic tradition is strong and birth control is discouraged. They wanted more tools for protection during oral sex: *“We obviously know you can get diseases through the vagina and penis but do not recognize that also from the mouth, it can be dangerous”* (teen focus group). Participants emphasized the value of practical, skill-based content. For example:

*Bring in condoms. Bring in things they can look at, models, things they can physically touch. I’ve taught sex education at middle school. They’re curious, ‘What is this for?’ And some of the women too, they have never experienced it* (Key Informant).

##### 3.2.3.1 Curriculum modifications

We made minor revisions to existing IMARA content on external condoms, internal condoms, and dental dams for language (e.g., replaced “female condom” with “internal condom”) and clarity (e.g., separated dental dams into its own activity). We retained the practical application activities from IMARA (e.g., putting an external condom on a penis model) and added an opportunity for practice using internal condoms. We procured contraception demonstration kits for teens and caregivers to also get hands on experience with contraception methods (Appendix 2).

#### 3.2.4 Session 3: Family strengthening

Participants spoke about the central role of family in Latine culture, reflecting *familismo*—a form of familial solidarity characterized by strong feelings of attachment, loyalty, and responsibility toward family members (40). They described how despite these strong bonds, Latine families feel uncomfortable talking about SRH and related topics (e.g., mental health, IPV), even when teens get along well with their parents. Sex is a taboo topic within families and is considered *“dirty”* (staff focus group). Teens and caregivers described the challenges of talking with each other about sex as follows:

*If I were to ask my parents, they would assume that I’m already [sexually] active* (Teen focus group).

*I get nervous because it’s a difficult subject* (Caregiver focus group).

*Since they [the teens] get defensive, we almost always end up angry* (Caregiver focus group).

Interlinked with these taboo topics are pressures Latina teens face from their families to act appropriately and maintain the image of the family. Teens also described expectations to respect their elders at all times and avoid any forms of confrontation:

*There is a lot of pressure on females to maintain the honor in their family. If something bad happens, it stigmatizes the family and causes a lot of friction* (Staff focus group).

*You have to respect older people when they do not respect you. They say a lot of mean things to you, but you are still forced to speak to them nicely and not correct them for their mistakes* (Teen focus group).

Participants expressed a desire for a program that facilitates learning and discussions between Latina teens and their mothers about sex, relationships, and related topics—i.e., *“…a time and a place where the mother and Latina daughter would feel comfortable talking about these things”* (teen focus group). They requested information on communication strategies for teens and caregivers and opportunities to practice how to communicate with each other.

##### 3.2.4.1 Curriculum modifications

We made minor revisions to IMARA content on forms of communication (i.e., passive, aggressive, and assertive forms) for clarity. We added an activity where teens and caregivers take turns sharing their experiences being a teen/caregiver, their views on family expectations in Latine culture, and their ideas for how their teen/caregiver can support and talk with each other openly and honestly (Appendix 3).

#### 3.2.5 Session 4: Gender and relationships

Participants spoke at length about different expectations of men and women in Latine culture, interlinked with concepts of *machismo* and *marianismo*. While these concepts have multiple definitions, *machismo* is generally believed to refer to a traditional role for masculinity in Latine communities. It can be characterized by toughness, aggressiveness, controlling behaviors, and womanizing (41). It can also be characterized as being courageous and hardworking, enduring strife to protect and provide for his family and reach his potential within a code of chivalry (42). Conversely, *marianismo* is the expectation for Latina women to fit into a submissive feminine role and exhibit virtue, humility, spirituality, self-sacrifice, and non-sexuality (41).

Study participants described how women are held to a different standard than men. They are expected to be modest, remain virgins and avoid pregnancy until married, pleasure their male partner sexually, and never get divorced, even if experiencing IPV. These expectations are instilled early and shaped by Catholic beliefs which ensure that *“really traditional ways of thinking are internalized”* (Staff focus group). Latina teens, in turn, said they face pressures to sexually please their boyfriends and conform with a society in which toxic relationships and IPV are normalized.

*I feel like nowadays, toxic people in a relationship is like romanticized. I want a toxico or I want a crazy guy* (Teen focus group).

*Women get accustomed to a lot of berating and making females feel small, and that’s passed down from generation to generation* (Staff focus group).

Latina teens are more likely to engage in risky sexual behavior and be in unhealthy or violent relationship even if they do not want to because, *“This is the belief system. ‘I know what is expected of me as a Latino girl’*” (Staff focus group). In addition to addressing gender norms in Latino culture in the curriculum, participants described the importance of interlinking gender identity and sexual orientation, which are also taboo topics in Latine households.

##### 3.2.5.1 Curriculum modifications

We made minor modifications to how IMARA content on healthy versus unhealthy relationships is delivered (i.e., in groups versus through individual work). We retained one partner communication role play and removed three others due to time limitations. We developed new content on consent, including sexual consent. We added activities and discussions addressing gender identity and sexual orientation, toxic relationships, and gender norms in Latine culture (Appendix 4).

#### 3.2.6 Additional adaptations

##### 3.2.6.1 Population

Study participants spoke about the importance of giving teens the option to participate with another female caregiver (e.g., a sister, aunt, or grandmother), since not all teens may want to participate with their mothers. For instance, a key informant explained, “*Who’s the second person involved besides your mother who you would feel more comfortable with? It’s gotta be an adult who’s also involved in your everyday life.*” As a result, we retained the ability for teens to choose which female caregiver they would like to participate with from IMARA but broadened the description of the program to a “teen-caregiver” rather than “mother-daughter” program.

##### 3.2.6.2 Duration

A key informant from the IMARA team shared that the duration of the IMARA program had been a challenge: *“A common complaint was that things are just too long. Especially for the girls after lunch, they would get sleepy.”* Thus, to support implementability, we allocated two hours per session for an eight-hour curriculum in total. This required eliminating some IMARA content, though we tried to retain the spirit of what was being conveyed in these activities. For example, while we removed IMARA content on teen/caregiver values and “getting to know you,” we added opportunities for teens and caregivers to informally reflect on each session at the end and to better understand each other’s perspectives in the Family Strengthening session. Additionally, we removed some topics that were not brought up during our qualitative data collection and were perceived by our CBPR team as being of lower priority (e.g., how women and girls are perceived in the media). Appendix 5 provides a detailed overview of IMARA activities that were retained and removed.

##### 3.2.6.3 Structure

Participants recommended structuring each session so that teens and caregivers start together in a large group to introduce the session outline, then move to separate spaces to cover content, and finally come back together for shared activities/discussion. The use of separate spaces was viewed as giving teens and caregivers a chance to learn in an open environment without judgment before addressing topics together, with joint sessions facilitating *“a meeting of the minds”* (Staff focus group). For example:

*Allow the daughters to have a space where they can freely speak and ask about sexual intercourse or the different protections, because if the moms hear the teens asking about condoms, they might see it as a bad thing* (Teen focus group).

*A lot of the topics will be new to moms too. I think there is benefit in having separate meetings with moms first to give them the tools and knowledge beforehand and then bringing girls in afterwards* (Staff focus group).

In response, we customized IMARA’s use of separate and joint activities to fit with a four-session structure. Specifically, we structured each session to begin with a joint introduction and activity, move to separate spaces for the bulk of the content, and come back together for joint activities/discussions. We added a consistent introductory activity and wrap-up for each session, including an opportunity for teens and caregivers to debrief in their pairs and a summary of key messages from the session.

##### 3.2.6.4 Group size and facilitators

We made minor modifications to activities to ensure that the content could be delivered to smaller groups of dyads (~3–6 dyads per workshop) by two facilitators (one with teens, one with caregivers) to support implementability and efficiency. Community organizations—the planned implementation site for the program—were unable to staff four facilitators per workshop.

##### 3.2.6.5 Culture

Participants highlighted the importance for the curriculum to be culturally tailored to Latinas so as to speak directly to their needs and experiences. We incorporated *surface adaptations* [i.e., customizing materials and messaging to ‘observable’ characteristics (43)], for instance, by ensuring that the names of characters in role plays were common names in Latine culture. We also incorporated *deep adaptations* [i.e., a reflection on cultural, social, psychological, environmental, and historical factors influence health behaviors differently across racial/ethnic populations (43)]. For instance, we included activities that prompt discussions among teens and caregivers about gender and family norms and expectations in Latine culture and their influence on teens’ sexual behavior.

While participants recognized the diversity of the Latine community, they felt that the curriculum content could be relevant to all Latine cultural groups. A staff focus group participant explained, *“[The Latine community] is very diverse, but I think that those cultural norms of how Latino families have been raised bridge a lot of those cultures, from a Mexican culture to a Dominican culture.”* Based on this feedback, we sought to ensure that all curriculum content would resonate with the Latina experience, broadly, but did not make modifications that would be specific to any cultural or geographic group within the Latine population.

##### 3.2.6.6 Language

In addition to language adaptations relating to culture, a key informant recommended ensuring gender inclusive language: *“I think there are pretty simple, not simplistic, but simple ways to shift language that might be more inclusive.”* We carefully reviewed the language to ensure that it would be gender inclusive. For example, we sought to avoid gendering anatomy by referring to “Anatomy of people with a vagina/penis” on handouts. We further ensured that scenarios would apply to all types of sexual relationships beyond heterosexual relationships. We generated curricula in English and Spanish based on feedback that teens would likely prefer English and caregivers Spanish.

##### 3.2.6.7 Where adaptations were not needed

A Latina key informant with experience in curriculum adaptation noted that some content may not need adaptation since IMARA, like Floreciendo, was designed for a minoritized population: “*Sometimes, there’s not much to adapt. When I think about Black communities and Latine communities, I feel like there are more similarities than there are differences. Both communities are marginalized in many areas.”* We therefore considered areas where content could remain unchanged. For example, we decided that the role play scenarios in the Family Strengthening session did not require changes since the cultural context was perceived as similar for Black and Latine families. More specifically, we kept a role play in which a mother is going through her daughter’s things in her daughter’s room, since our CBPR team noted that issues of privacy are considered a hot button topic in Latine homes.

## 4 Discussion

Many frameworks exist to guide program adaptation, which has gained notable interest in the implementation science field (3). However, literature is lacking in descriptions of adaptation processes (1–3). In this study, we articulated how we used qualitative data to systematically adapt IMARA for Latinas and documented our adaptations using the FRAME (1). Our study thus adds to the growing literature on examples of how to use the FRAME in practice (44, 45). Our paper further provides considerable detail on the content included in the Floreciendo and IMARA programs, which could inform future program development and adaptation in the field of adolescent sexual and reproductive health. We believe that sharing curriculum content and materials, where possible, will strengthen the quality and science of programming.

Our study answers calls in the implementation science literature for clarity on the processes through which interventions are adapted (1, 2), greater transparency in adaptation reporting (1), a need for more “practice-based evidence” emphasizing insights from practitioners and lived experiences of relevant populations (46), and the use of community-engaged research and CBPR approaches to enhance program adaptation processes (47, 48). Our adaptation process, carried out using CBPR, aligns with recommendations in the literature for how to adapt an intervention to a new setting or population (3). In this paper, informed by Escoffery’s recommended adaptation steps (3), we illustrate how our content-level adaptations were driven by feedback from Latina teens, female caregivers of Latina teens, staff from community organizations who will implement Floreciendo, and other experts, including the IMARA program originator. By making adaptations specific to the needs, language, values, and norms of Latinas, we hope to enhance Floreciendo’s potential of addressing SRH-related disparities among Latina teens (9–11) and strengthen efforts to achieve health equity for the Latine community (2, 47).

The findings presented here played a key role in informing our next steps for Floreciendo. Building on these findings, our team conducted additional data collection to understand the potential program implementation, including workshop logistics, barriers and facilitators, strategies, and potential for adoption and sustainability in community organizations (49). To move toward optimization within the MOST framework (37), we decided that the Foundations in Sexual Risk Prevention session would be a “constant” session that all participants would receive, and we would test the effectiveness of the remaining three sessions in an optimization trial. We subsequently created a conceptual model for how the four sessions would lead to our desired outcomes. This conceptual model will serve as the basis of our testing of Floreciendo, first through theater testing, then in a pilot optimization trial, and later in a fully powered optimization randomized controlled trial.

Study limitations must be acknowledged. Most teen and caregiver focus group participants identified as being of Mexican descent, whereas our goal was to adapt the program to fit all Latinas. However, organizational staff contributed feedback from a wide range of Latin American origins and participants expressed their belief that comments in the focus groups would be applicable to all Latinas. Additionally, one focus group with organization staff took place much later than the bulk of the data collection, although findings reinforced themes from the early focus groups and ensured the inclusion of insights from a new implementing partner organization. Finally, the eligibility criteria for teens required proficiency in English language due to language constraints of the study team. This could have limited the breadth of perspectives received, but our community partners indicated that the vast majority of Latina teens they work with speak English so this is unlikely to have notably impacted our sample.

In conclusion, in this paper, we presented our systematic adaptation process and our meticulous documentation of our adaptations using the FRAME. We underscore the importance of transparency in adaptation processes for rigor and reproducibility and to strengthen our collective understanding of the impact of adaptations on desired program outcomes. These adaptations will support our goals of strengthening Latina teens’ SRH outcomes and enhancing efforts to achieve health equity. Our findings further directly informed next steps for Floreciendo using the MOST framework, including through additional exploration of implementation considerations, theater testing the adapted intervention, a pilot optimization trial, and an optimization randomized controlled trial.

## Data Availability

The raw data supporting the conclusions of this article will be made available by the authors, without undue reservation.

## References

[1] Stirman S Baumann A Miller C. The FRAME: an expanded framework for reporting adaptations and modifications to evidence-based interventions. Implement Sci. (2019) 14:58. doi: 10.1186/s13012-019-0898-y, PMID: 31171014 PMC6554895

[2] Baumann A Cabassa L Stirman S. Adaptation in dissemination and implementation science In: Dissemination and implementation research in health: translating science to practice, vol. 2. Oxford: Oxford University Press (2017). 286–300.

[3] Escoffery C Lebow-Skelley E Udelson H Böing EA Wood R Fernandez ME. A scoping study of frameworks for adapting public health evidence-based interventions. Transl Behav Med. (2019) 9:1–10. doi: 10.1093/tbm/ibx067, PMID: 29346635 PMC6305563

[4] Hall GCN Ibaraki AY Huang ER Marti CN Stice E. A meta-analysis of cultural adaptations of psychological interventions. Behav Ther. (2016) 47:993–1014. doi: 10.1016/j.beth.2016.09.005 27993346

[5] Bernal G Jiménez-Chafey MI Domenech Rodríguez MM. Cultural adaptation of treatments: a resource for considering culture in evidence-based practice. Prof Psychol Res Pract. (2009) 40:361–8. doi: 10.1037/a0016401

[6] Cabassa LJ Baumann AA. A two-way street: bridging implementation science and cultural adaptations of mental health treatments. Implement Sci. (2013) 8:1–14. doi: 10.1186/1748-5908-8-90 23958445 PMC3765289

[7] Baumann A Shelton R Kumanyika S Haire-Joshu D. Advancing healthcare equity through dissemination and implementation science. Health Serv Res. (2023) 58:327–44. doi: 10.1111/1475-6773.14175, PMID: 37219339 PMC10684051

[8] Braveman P. What are health disparities and health equity? We need to be clear. Public Health Rep. (2014) 129:5–8. doi: 10.1177/00333549141291S203, PMID: 24385658 PMC3863701

[9] CDC. (2020). National Center for HIV/AIDS, Viral Hepatitis, STD, and TB Prevention: Atlas plus. Available online at: https://www.cdc.gov/nchhstp/atlas/index.htm?s_cid=bb-od-atlasplus_002

[10] CDC. Youth risk behavior survey: Data Summary & Trends Report: 2007–2017. Atlanta, GA: National Center for HIV/AIDS, Viral Hepatitis, STD, and TB Prevention (2018).

[11] Martin J Hamilton B Osterman M. Births in the United States, 2017. NCHS Data Brief. (2018):1–8. 30156535

[12] SAMHSA. (2018) National Survey on drug use and health: Hispanics, Latino or Spanish origin or descent 2020. Available online at: https://www.samhsa.gov/data/report/2018-nsduh-hispanics-latino-or-spanish-origin-or-desce

[13] Smith SG Basile KC Gilbert LK Merrick MT Patel N Walling M. (2017) National Center for Injury Prevention and Control (U.S.). Division of Violence Prevention.

[14] Panchal N. (2024). Recent trends in mental health and substance use concerns among adolescents. Available online at: https://www.kff.org/mental-health/issue-brief/recent-trends-in-mental-health-and-substance-use-concerns-among-adolescents/

[15] Duby Z McClinton Appollis T Jonas K Maruping K Dietrich J LoVette A. “As a young pregnant girl … the challenges you face”: exploring the intersection between mental health and sexual and reproductive health amongst adolescent girls and young women in South Africa. AIDS Behavior. (2012) 25:344–53. doi: 10.1007/s10461-020-02974-3, PMID: 32683636 PMC7368608

[16] Rosen B Dauria E Shumway M Smith J Koinis-Mitchell D Tolou-Shams M. Association of pregnancy attitudes and intentions with sexual activity and psychiatric symptoms in justice-involved youth. Child Youth Serv Rev. (2022) 138:106510. doi: 10.1016/j.childyouth.2022.106510, PMID: 38107676 PMC10723635

[17] Seth P DiClemente R Lovvorn A. State of the evidence: intimate partner violence and HIV/STI risk among adolescents. Curr HIV Res. (2013) 11:528–35. doi: 10.2174/1570162X12666140129103122, PMID: 24476354

[18] Ritchwood T Ford H DeCoster J Sutton M Lochman J. Risky sexual behavior and substance use among adolescents: a meta-analysis. Child Youth Serv Rev. (2015) 52:74–88. doi: 10.1016/j.childyouth.2015.03.005, PMID: 25825550 PMC4375751

[19] Guilamo-Ramos V Dittus P Jaccard J Goldberg V Casillas E Bouris A. The content and process of mother—adolescent communication about sex in Latino families. Soc Work Res. (2006) 30:169–81. doi: 10.1093/swr/30.3.169

[20] Donenberg G Kendall A Emerson E Fletcher F Bray B McCabe K. IMARA: a mother-daughter group randomized controlled trial to reduce sexually transmitted infections in adolescent African American women. PLoS One. (2020) 15:e0239650. doi: 10.1371/journal.pone.0239650, PMID: 33137103 PMC7605636

[21] Donenberg G. (2024). IMARA (informed, motivated, aware, and responsible about AIDS) compendium of evidence-based interventions and best practices for HIV prevention: CDC. Available online at: https://wwwn.cdc.gov/HIVCompendium/SearchInterventions

[22] Hutchinson M. The influence of sexual risk communication between parents and daughters on sexual risk behaviors. Fam Relat. (2002) 51:238–47. doi: 10.1111/j.1741-3729.2002.00238.x

[23] Meneses L Orrell-Valente J Guendelman S Oman D Irwin C. Racial/ethnic differences in mother-daughter communication about sex. J Adolesc Health. (2006) 39:128–31. doi: 10.1016/j.jadohealth.2005.08.005, PMID: 16781975

[24] Becerra R de Anda D. Pregnancy and motherhood among Mexican American adolescents. Health Soc Work. (1984) 9:106–23. doi: 10.1093/hsw/9.2.106, PMID: 6724424

[25] Zayas L Solari F. Early childhood socialization in Hispanic families: context, culture, and practice implications. Prof Psychol Res Pract. (1994) 25:200–6. doi: 10.1037/0735-7028.25.3.200

[26] Guilamo-Ramos V Bouris A Jaccard J Gonzalez B McCoy W Aranda D. A parent-based intervention to reduce sexual risk behavior in early adolescence: building alliances between physicians, social workers, and parents. J Adolesc Health. (2011) 48:159–63. doi: 10.1016/j.jadohealth.2010.06.007, PMID: 21257114 PMC3118646

[27] Afable-Munsuz A Brindis CD. Acculturation and the sexual and reproductive health of Latino youth in the United States: a literature review. Perspect Sex Reprod Health. (2006) 38:208–19. doi: 10.1363/3820806, PMID: 17162313

[28] Alarcão V Stefanovska-Petkovska M Virgolino A Santos O Costa A. Intersections of immigration and sexual/reproductive health: an umbrella literature review with a focus on health equity. Soc Sci. (2021) 10:63. doi: 10.3390/socsci10020063

[29] Martinez O Wu E Moya E Chavez Baray S Dodge B Shultz Z. Overcoming issues and challenges in serving the sexual health needs of Latino immigrants in the United States. Health Education Monograph Series. (2014) 31:43–51.

[30] Israel B Eng E Schulz A Parker E. Methods in community-based participatory research for health. Hoboken, NJ: John Wiley & Sons (2005).

[31] Corlett S Mavin S. (2018). The SAGE handbook of qualitative business and management research methods: History and traditions. London: SAGE. 377–398.

[32] Donenberg GR Pao M. Understanding HIV/AIDS: psychosocial and psychiatric issues in youths. Contemporary Psychiatry. (2003) 2:1–8. PMID: 25364309 PMC4213805

[33] DiClemente RJ Wingood GM. A randomized controlled trial of an HIV sexual risk-reduction intervention for young African-American women. JAMA J Am Med Assoc. (1995) 274:1271–6. doi: 10.1001/jama.1995.03530160023028, PMID: 7563531

[34] DiClemente RJ Wingood GM Harrington KF Lang DL Davies SL Hook EW. Efficacy of an HIV prevention intervention for African American adolescent girls: a randomized controlled trial. JAMA J Am Med Assoc. (2004) 292:171–9. doi: 10.1001/jama.292.2.171, PMID: 15249566

[35] Brown L Hadley W Donenberg G DiClemente R Lescano CM Lang D. Project STYLE: a multisite RCT for HIV-prevention among youths in mental health treatment. Psychiatr Serv. (2014) 65:338–44. doi: 10.1176/appi.ps.201300095, PMID: 24382603 PMC9215702

[36] Miller CJ Barnett ML Baumann AA Gutner CA Wiltsey-Stirman S. The FRAME-IS: a framework for documenting modifications to implementation strategies in healthcare. Implement Sci. (2021) 16:1–12. doi: 10.1186/s13012-021-01105-3, PMID: 33827716 PMC8024675

[37] Collins L. Optimization of behavioral, biobehavioral, and biomedical interventions: The multiphase optimization strategy (MOST). Cham: Springer International Publishing (2018).

[38] Saldana J. The coding manual for qualitative researchers. Los Angeles: SAGE (2013).

[39] Tobin G Begley C. Methodological rigour within a qualitative framework. J Adv Nurs. (2004) 48:388–96. doi: 10.1111/j.1365-2648.2004.03207.x, PMID: 15500533

[40] Gonzales SM. Cultivating familismo: belonging and inclusion in one Latina/o learning community. Int J Incl Educ. (2019) 23:937–49. doi: 10.1080/13603116.2019.1602362

[41] Garcia E. Blending the gender binary: the machismo-marianismo dyad as a coping mechanism. Honors Project. (2021)

[42] Coronado JD. (2018). "I'm not Gonna die in this damn place": Manliness, identity, and survival of the Mexican American Vietnam prisoners of war. East Lansing, MI: Michigan State University Press. 39–56.

[43] Resnicow K Baranowski T Ahluwalia JS Braithwaite RL. Cultural sensitivity in public health: defined and demystified. Ethn Dis. (1999) 9:10–21. PMID: 10355471

[44] Chhun N Oketch D Agot K Mangale DI Badia J Kibugi J. Using FRAME to characterize provider-identified adaptations to a stepped care intervention for adolescents and youth living with HIV in Kenya: a mixed methods approach. J Int AIDS Soc. (2024) 27:e26261. doi: 10.1002/jia2.26261, PMID: 38965971 PMC11224585

[45] Abraham J Meng A Baumann A Holzer KJ Lenard E Freedland KE. A multi-and mixed-method adaptation study of a patient-centered perioperative mental health intervention bundle. BMC Health Serv Res. (2023) 23:1175. doi: 10.1186/s12913-023-10186-3, PMID: 37891574 PMC10612159

[46] Ammerman A Smith TW Calancie L. Practice-based evidence in public health: improving reach, relevance, and results. Annu Rev Public Health. (2014) 35:47–63. doi: 10.1146/annurev-publhealth-032013-182458, PMID: 24641554

[47] Baumann A Cabassa L. Reframing implementation science to address inequities in healthcare delivery. BMC Health Serv Res. (2020) 20:1–9. doi: 10.1186/s12913-020-4975-3, PMID: 32164706 PMC7069050

[48] Wallerstein N Duran B. Community-based participatory research contributions to intervention research: the intersection of science and practice to improve health equity. Am J Public Health. (2010) 100:S40–6. doi: 10.2105/AJPH.2009.184036, PMID: 20147663 PMC2837458

[49] Merrill KG Silva J Sedeño A Salgado S Vargas S Cano J. Preparing to implement Floreciendo with Latina teens and their female caregivers: Integrating implementation science and the MOST framework. Transl. Behav. Med. in press. (2025). 10.1093/tbm/ibaf005 PMC11886812 40052537

