# Supplementary material for: Adapting a sexual and reproductive health program for Latina teens and their female caregivers: a qualitative study
Source: Front Public Health. 2025 Feb 19;13:1501757. doi: 10.3389/fpubh.2025.1501757 (PMC11879970; doi:10.3389/fpubh.2025.1501757)
Supplement: Supplementary file 1 [file Data_Sheet_1.pdf]

Appendix 1: Curriculum adaptations to the Foundations in Sexual Risk Prevention session of Floreciendo, reported using the FRAME

**Floreciendo activity**	**IMARA activity(ies) adapted from**	**Nature of modification**	**Description of modification**	**Feedback from WHOM informed the adaptation?**	**Representative quotes**
Welcome & Introduction	Greeting & Icebreaker; IMARA Program Introduction; Ground Rules	Adding elements/ Tailoring/ Refining	Customized program intro. Condensed ground rules and pact. Added a note that participants should talk about what they are learning with father figures at home.	Staff, Key informants	"Sometimes, when we don't have dads in the room, they're still present in the sense that we're still talking about them and thinking about them. How will they show up in the conversation?" (Key informant)
Mental health & the feeling thermometer	Feeling Thermometer / Affect Management	Adding elements/ Refining	Added content on what mental health is and discussion about other strategies for emotional regulation beyond the Feeling Thermometer activity. Updated handout/poster for clarity and to add Spanish translations.	Teens, Staff, Key informant	"[Include in the curriculum] a conversation about what does mental health mean to you, …stigma, accessing therapy...." (Staff focus group)
Young, Latina, and Female	Young, Black, & Female; Why Don't I Use Condoms?	Adding elements/ Tailoring/ Refining	Customized for Latinas by including content on sexual decision-making specifically for Latina teens. Added content on sexual pleasure and masturbation.	Teens, Caregivers, Staff, Key informant	"[Address] the importance of exploring yourself...Our culture points that how dirty is that that a woman touches herself....It's very important to teach those things and talk about it in a normal way..." (Staff focus group)
Virus Carrier Handshake	Virus Carrier Handshake	Adding elements/ Refining	Broadened to STIs beyond HIV specifically. Added a "mutually faithful" role to the activity. Clarified activity steps. Added discussion about STI prevention.		
True or False	HIV/AIDS Information, Motivation, Skills	Adding elements	Added a true/false game to cover facts about HIV, condoms, birth control, and HPV and to dispel myths about using tampons. Removed video about HIV/AIDS.	Teens, Caregivers, Staff, Key informants	"[Caregivers] would tell girls not to use tampons because tampons is going to make you want to have sex." (Staff focus group)<br><br>"Let them know what is HPV and the importance of getting the vaccine at a young age" (Key informant)

**Floreciendo activity**	**IMARA activity(ies) adapted from**	**Nature of modification**	**Description of modification**	**Feedback from WHOM informed the adaptation?**	**Representative quotes**
High, Low, or No Risk	High, Low, No Risk	Tailoring/ Rewording/ Refining / Removing elements	Simplified wording for clarity. Added definitions for terms like "mutual masturbation." Replaced risky situations involving drug use with alcohol use.	Teens, Staff, Key Informants	"A lot of these moms don't know the [sexual] risks because they were never taught" (Key informant, Latina CHW)
Parental Monitoring (Caregivers)	Adolescent Development; Promoting Parental Monitoring	Rewording/ Refining	Made minor updates to the structure for clarity.	Teens	"Privacy, trust of daughters from mothers" (Teen focus group)
Personalizing Risky Situations (Teens)	Identifying & Personalizing HIV/AIDS Risk Situations	Refining	Modified sharing of risky situation to be a personal story from the facilitator.		
Pair & Share	None	Adding elements	Add an opportunity for teens and caregivers to debrief on the topics of the day		
Key Messages	None	Adding elements	Added a review of key messages from the session		
Supplemental material/ handouts	Handouts	Adding elements/ Tailoring/ Rewording/ Refining	Added handout on anatomy. Updated examples in Parental Monitoring worksheet. Customized resources.	Teens, Staff, Key informants	"The vagina- it's like, 'Okay, what is that?' Sometimes people want to ask" (Staff focus group)<br><br>"Learning about body parts helps us respect and honor them" (Key informant)

Appendix 2: Curriculum adaptations to the Condoms & Contraception session of Floreciendo, reported using the FRAME

Floreciendo activity	IMARA activity(ies) adapted from	Nature of modification	Description of modification	Feedback from WHOM informed the adaptation?	Representative quotes
Welcome & Introduction	Greeting & Icebreaker	Tailoring	Customized overview for this session		
External Condoms (LIPSTICK)	LIPSTICK	Rewording/ Refining	Changed wording from "condom" to "external condom." Customized discussion topics about the activity (e.g., added discussion about what to do if the external condom is put on inside out). Updated the handout/poster for visual appeal.	Teens, Staff, Key informants	"There is a condom for everyone, but there are many options and the options that fit best for me, they're not the same as for her. We need more information about that." (Staff focus group)<br><br>"How to read the label of a condom…" (Key informant)
Internal Condoms	Female Condom Use	Rewording/ Refining/ Adding elements	Changed wording from "female condom" to "internal condom." Incorporated practice of how to use a female condom. Updated handout/poster.	Teens, Staff, Key informants	"We don't know too much about the female condom…I was like, 'Does it work like that?'" (Teen focus group)
Dental Dams	None	Refining/ Adding elements	Separated content into its own activity (from LIPSTICK). Added a handout of steps to use a dental dam.	Teens, Caregivers, Staff	"[Oral sex] it's become more common and normalized with the younger generation…The mothers don't really talk about that with their daughters." (Teen focus group)
Do's and Don'ts of Condom and Dental Dam Use	None	Refining	Separated content into its own activity and clarified the do's and don'ts using lists.		
Contraception	None	Adding elements	Created new session. Participants can touch/feel various contraceptive methods while learning about them.	Teen, Staff, Key informants	"I think [the program] can help Latina teens with how to use products. Most of us don't actually know how to use it, like birth control" (Teen focus group)
Teen-Caregiver LIPSTICK	LIPSTICK Rehearsal	Adding elements / Refining	Integrated a game when teens and caregivers practice putting on condoms together.		

Floreciendo activity	IMARA activity(ies) adapted from	Nature of modification	Description of modification	Feedback from WHOM informed the adaptation?	Representative quotes
Pair & Share	None	Adding elements	Added an opportunity for teens and caregivers to debrief on the topics of the day		
Key Messages	None	Adding elements	Added a review of key messages from the session		
Supplemental material/handouts	Handouts	Tailoring/ refining/Adding elements	Created handouts with instructions on using dental dams and internal condoms and information about forms of contraception. Updated the design of the LIPSTICK and "how to talk about condoms with a partner" handouts.		

Appendix 3: Curriculum adaptations to the Family Strengthening session of Floreciendo, reported using the FRAME

**Floreciendo activity**	**IMARA activity(ies) adapted from**	**Nature of modification**	**Description of modification**	**Feedback from WHOM informed the adaptation?**	**Representative quotes**
Welcome & Introduction	Greeting & Icebreaker	Refining	Customized overview for this session	N/A	N/A
Passive, Aggressive, and Assertive Communication (including separate assertive communication role-play)	Assertive Communication with Mothers/ Daughters; Role Play Assertive Communication	Refining / Removing elements	Provided an opportunity for teens and caregivers to practice role plays in pairs within their separate groups. Removed one role play for time.	Teens, Caregivers, Staff, Key informants	"[The curriculum should address] the different types of communication, because it's important…We didn't communicate with our parents about it. We would find somebody else to communicate it." (Staff focus group)<br>“For me it would be… preparing us moms, share tips on how to not get mad to help us talk with our teen.” (Caregiver focus group)
Joint Assertive Communication Role play	Role Play Assertive Communication	Refining	Teens and caregivers role play in their own roles rather than reversing roles (where teens act as the caregiver and vice versa)	Key informant	"In IMARA, I feel like the role plays were always very well received. People really enjoyed them" (Key informant)
Family Norms and Expectations	Mother-Daughter Values Discussion; Getting to know you	Adding elements / Tailoring	Added an activity for teens and caregivers to share their perspectives on being a Latina teen/caregiver, challenges they face, and how they would like to communicate, using inner and outer circles	Teens, Caregivers, Staff, Key informants	“My mom's hard-headed. It's very hard to talk about feelings. It's either her way or the highway. If she doesn't like something, she immediately shuts down the conversation. I think it's more about having that openness between both parties, both daughter and mother.” (Teen focus group)
Pair & Share	None	Adding elements	Add an opportunity for teens and caregivers to debrief on the topics of the day and give each other praise		

Floreciendo activity	IMARA activity(ies) adapted from	Nature of modification	Description of modification	Feedback from WHOM informed the adaptation?	Representative quotes
Key Messages	None	Adding elements	Added a review of key messages from the session		
Supplemental Materials / Handouts	Handouts	Refining	Replaced handouts on passive, aggressive, and assertive communication with a single handout called “Saying it assertively.”		

Appendix 4: Curriculum adaptations to the Gender & Relationships session of Floreciendo, reported using the FRAME

Floreciendo activity	IMARA activity(ies) adapted from	Nature of modification	Description of modification	Feedback from WHOM informed the adaptation?	Representative quotes
Welcome & Introduction	Greeting & Icebreaker	Refining	Customized overview for this session		
Our identities	None	Adding elements	Added content on sexual orientation and gender identities.	Staff, Teens	"[The curriculum should address] sexual orientation and gender identity" (Staff)<br>"The LGBTQ+ community- that is less talked about because sometimes our parents don't like it" (Teen)
Healthy/ unhealthy relationships	What do healthy & unhealthy relationships look like?	Refining/ Removing elements	Changed activity so that participants decide whether relationship scenarios are healthy or unhealthy in small groups rather than individually. Removed activity about types of partners. Retained one role play and removed three role plays for time.	Staff, Teens, Key informants	"I think there might be levels of toxicity. There's some emotional abuse and physical abuse, all of that." (Teen)<br>"They might not know what a healthy relationship looks like because they don't have that role model" (Key informant)
KISS- Keep It Simple Sister	KISS- Keep It Simple Sister; Assertive Communication with Partners	Refining	Made minor updates to the activity flow for clarity.	Staff	"Sometimes, boys say to girls, 'If you don't have sex with me, I'm going to have sex with a Guatemalan person.' That affects the girls. It's like, "I better have sex with him.'" (staff)

Floreciendo activity	IMARA activity(ies) adapted from	Nature of modification	Description of modification	Feedback from WHOM informed the adaptation?	Representative quotes
Consent and Partner Violence	What does abuse look like?	Adding elements/ Tailoring/ Refining	Changed wording from “abuse” to “violence.” Integrated content on violence with new content on consent and sexual consent.	Staff, Teens	“Some mothers need to learn that consent should be a very big thing—especially because some of them have sex without consent…Rape can still happen between partners." (Teen focus group)<br><br>“...I feel like it's really important that you express what it is to be in a violent relationship between couples, because it's not just always physical. It's sometimes controlling you or telling you you can't do things when you can …” (Staff focus group)
Gender norms and relationships in Latino Culture	Expands on IMARA’s use of poems, songs, and other culturally-specific content	Adding elements / Tailoring	Added a discusión about gender roles and expectations in Latine culture and their impact on romantic relationships. Added an activity to listen to a popular song, El Toxico, and discuss the lyrics as they relate to healthy / unhealthy relationships.	Staff, Teens	"I feel like nowadays, toxic people in a relationship is like romanticized. I want a toxico or I want a crazy guy or something. I feel like these days with social media it's starting to get romanticized." (Teen)
Pair & Share	None	Adding elements	Add an opportunity for teens and caregivers to debrief on the topics of the day		
Key Messages	None	Adding elements	Added a review of key messages from the session		
Supplemental material/ handouts	Handouts	Adding elements/ Refining/ Tailoring	Updated the handout on partner communication, updated IPV resources handout, refined the handout on IPV, added a handout for ‘El Toxico’ activity, and added a handout on sexual consent.	Teens	“[The curriculum should address] how to prevent toxic relationships or the resources that exist [for IPV]” (Teen focus group)

Appendix 5: IMARA curriculum topics and whether they were retained (in green) or removed (in red)

Day 1: Afternoon
Greeting & Icebreaker
IMARA Program Introduction
My Mothers, My Daughters
IMARA Motto & Ground Rules
The IMARA Pact
Get to Know You Game (1)
Assertive Communication with Mothers/Daughters
"Rephrase it" Game (Mothers only)
Role Play Assertive Communication
Media Masquerade
Young, Black, & Female
Successful African-American Female Role Models
Phenomenal Women
Virus Carrier Handshake
High, Low, No Risk
Values- What Matters Most
Get to Know You Game (2)
Reverse Role Play
Successful Black Women: Taboo Game
Public Service Announcements
Wrap Up & Homework

Day 2: Morning
Greetings & Icebreaker
Opening Poem and IMARA
Homework Review
Adolescent Development (Mothers only)
Promoting Parental Monitoring (Mothers only)
Feeling Thermometer/Affect Management
Identifying and Personalizing HIV/AIDS Risk Situations
Assertive Communication with Partners and Peers
Why Don't I Use Condoms?
KISS- Keep It Simple Sister
Mother Challenge (1) (Daughters only)
Introducing LIPSTICK
Female Condom Use
Debriefing LIPSTICK & Female Condoms
Mothers Talking to Daughters about Sex & Practice (Mothers only)
Thought Works
Mother Challenge (2)
Mother-Daughter Values Discussion
The Value of My Body
HIV/AIDS Information, Motivation, Skills Video

Day 2: Afternoon
Still I Rise
What do Healthy & Unhealthy Relationships Look Like?
Dating Game and Partner Types
Your Partner Choices and Your Daughter (Mothers Only)
Pieces & Parts
What Does Abuse Look Like?
Your Options
Icebreaker
De-Mystifying HIV Testing
Observed Mother-Daughter Communication
LIPSTICK Rehearsal
Alcohol & Sex
Taking Care of You
Graduation
Wrap Up & Evaluation

Notes: Activities in green were retained. Activities in red were removed. Activities are shared with permission by the creator of IMARA.
